# In Vitro Simulated Hemoperfusion on Seraph^®^-100 as a Promising Strategy to Counteract Sepsis

**DOI:** 10.3390/biomedicines12030575

**Published:** 2024-03-05

**Authors:** Antonio Lacquaniti, Antonella Smeriglio, Susanna Campo, Erminia La Camera, Giovanni Lanteri, Elena Giunta, Paolo Monardo, Domenico Trombetta

**Affiliations:** 1 Nephrology and Dialysis Unit, Department of Internal Medicine, Papardo Hospital, 98158 Messina, Italy; ant.lacq@gmail.com (A.L.);; 2 Department of Chemical, Biological, Pharmaceutical and Environmental Sciences, University of Messina, 98122 Messina, Italyerminia.lacamera@unime.it (E.L.C.);; 3 Pathology Unit, Papardo Hospital, 98158 Messina, Italy; elenagiunta@live.it

**Keywords:** acute kidney injury, adsorption, renal replacement therapy, sepsis, Seraph^®^-100

## Abstract

Blood purification represents a treatment option for sepsis, improving inflammation and the hyper-activated immune system. This study investigates the binding efficacy of Seraph^®^-100 against 10^8^ CFU/mL of *Staphylococcus aureus* (*S. aureus*), *Pseudomonas aeruginosa* (*P. aeruginosa*), and *Escherichia coli* (*E. coli*) during a simulated hemoperfusion treatment. The fluorescence-activated cell sorting (FACS) technique was used to evaluate the bacteria reduction, whereas kinetic analysis and cultures revealed bacterial detection and counting at established time points. At the end of the experiment, the filter was cut at three different levels, obtaining suspensions for cultures and scanning electron microscopy (SEM) analyses. The FACS technique revealed a 78.77% reduction of the total bacterial load at the end of the treatment, with maximum filter sequestration occurring in the first 30 min of the treatment. Non-linear regression analysis of kinetic experiments (T_0–240 min_) highlighted a lower growth rate of *S. aureus* than the other two Gram bacteria, demonstrating a greater affinity without influencing a reduction rate of 99% for all three bacteria. The analyses of the suspension aliquots of the filter sections confirmed these data, revealing 1 × 10^8^ CFU/mL, equal to the initial bacterial charge. Furthermore, the filter head adsorbed approximately 50% of bacteria, whereas the remaining amount was equally distributed between the body and the tail, as corroborated by SEM analysis. In conclusion, Seraph^®^-100 adsorbed 10^8^ CFU/mL of *S. aureus*, *E. coli*, and *P. aeruginosa* during an in vitro simulated hemoperfusion session.

## 1. Introduction

Sepsis, a complex and systemic disorder resulting from a dysregulated host response to an infection, leads to acute organ dysfunction and a high risk of death [1]. In 2020, about three million incident cases of sepsis were recorded in Europe [2], and the lung (64% of cases), followed by the abdomen (20%), bloodstream (15%), and renal and genitourinary tracts (14%), represented the most common sites of infection [3]. In particular, the Sepsis Occurrence in Acutely Ill Patients (SOAP) study reported a similar prevalence of Gram+ and Gram− bacterial infections among septic patients, with *Staphylococcus aureus*, *Pseudomonas* sp., and *Escherichia coli* being the most frequently identified organisms [4].

Behind epidemiological and clinical considerations, pharmacoeconomic implications are not negligible because they are related to the increased costs of sepsis when multidrug-resistant (MDR) bacteria occur [5,6]. In 2017, the World Health Assembly urged the World Health Organization member states to prioritize sepsis in their national health systems, recognizing sepsis as a global health priority [7]. Five years after this resolution, the challenge is always ongoing: facing the lack of standardized definitions or not applying them uniformly, reducing the delay of microbiological services to deliver blood culture results, and improving interdisciplinary collaboration. However, over the past decades, growing literature data have enhanced the timing of the diagnosis and treatment of sepsis, considering it not only an inflammatory disorder but highlighting an abnormal host response, triggering acute complications and organ failures [8,9].

**Figure 1:**
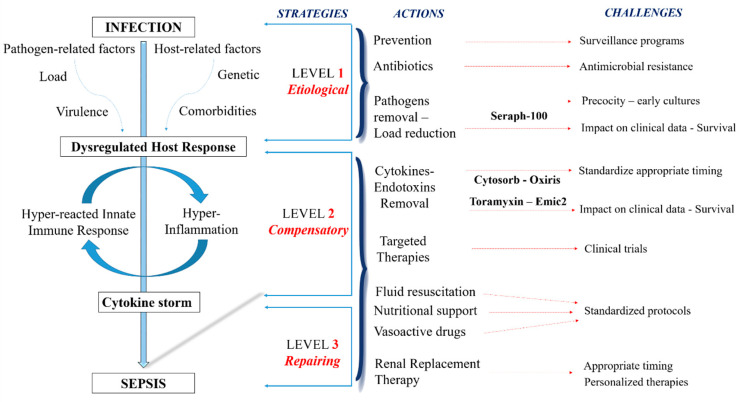
Strategies, actions, and challenges in septic patients.

In this perspective, a recent study defines sepsis as a syndromic entity, distinguishing “inflammopathic” or “coagulopathic” endotypes with the consequent personalization of treatment strategies [10]. Leaving aside this “research approach”, screening for signs and symptoms of sepsis and septic shock facilitates earlier identification and intervention in clinical practice, aiming at the efficient and early removal of endotoxins and inflammatory factors and optimizing patient therapies and outcomes [11]. Antibiotics, the most effective weapon against bacterial infections, are becoming less effective, and new molecules are responsible for high costs. Therefore, there is an urgent need for alternative treatment options. In recent years, continuous blood purification therapies have represented a treatment option for sepsis, removing inflammatory mediators and acting on the hyper-activation of the innate and acquired immune systems, reducing the negative systemic response [12,13]. Whereas a single targeted cytokine removal was unsuccessful, the unselective removal of cytokines by hemoperfusion using adsorber systems, such as CytoSorb, improved the outcomes in septic patients [14,15]. However, contrasting data should be underlined by analyzing several studies and meta-analyses, not revealing a reduction in mortality by treatment with this adsorber filter [16]. Conflicting reports refer to the oXiris hemofilter, a hydrogel structure of the AN69 membrane coated with polyethyleneimine and heparin that adsorbs endotoxins and inflammatory cytokines [17]. Recent data-related oXiris filters have improved mortality, sequential organ failure assessment (SOFA) scores, and intensive care unit (ICU) stays [18]. In this context, encouraging data are emerging about the Seraph^®^-100 Microbind Affinity Filter (Exthera Medical, Martinez, CA, USA) [19]. Whereas the absorber filters reduced inflammatory response mediators, lipopolysaccharide, and endotoxins, Seraph^®^-100, based on ultrahigh-molecular-weight polyethylene beads with end point-attached heparin, removes bacteria, fungi, and viruses irreversibly from the bloodstream through binding with the immobilized heparin and miming the interaction with heparan sulfate on the cell surface (Figure 1) [20].

To date, literature data refer to single-center experiences, often referring to the COVID-19 pandemic, complicated or not by sepsis, with positive effects on hemodynamic parameters and vasopressor requirements when this filter is applied [21,22]. Other results have been obtained in hemodialysis patients with catheter-related bacteremia, resulting in a faster resolution of bloodstream infections if treated with Seraph^®^-100 precociously within 66 h after the initial positive blood culture [23]. The correct timing for using this filter, whether applied alone or associated with continuous renal replacement therapy (CRRT), is an important topic. The preliminary results suggest “the sooner, the better”, considering that applying the Seraph^®^-100 filter after 60 h from ICU admission or bacterial infection is associated with poor outcomes [24]. Several studies analyzed the performances of this filter in vitro, testing its binding activity to several pathogens with not negligible bias due to the use of miniaturized micro-columns pre-treated with infected solutions [25] or analyzing the effects after single passage through a column packed with heparinized beads in a laboratory setting, far from clinical practice [26]. Other considerations could be obtained by extrapolating data from in vitro studies analyzing the effects of Seraph^®^-100 on drug pharmacokinetics. Seraph^®^-100 did not influence the clearance of several antibiotics, except for aminoglycosides, including vancomycin, gentamicin, meropenem, and imipenem, with consequent positive clinical implications [27]. However, further studies should evaluate and confirm if a drug dose adjustment is required during Seraph^®^-100 use, avoiding sub-therapeutic antibiotic levels, which negatively influence the success of clinical trials, sepsis treatment, and overall survival. Starting from these assumptions, sepsis can be caused by multiple microorganisms, and this study investigates the binding efficacy of Seraph^®^-100 against three common sepsis pathogens, such as *Staphylococcus aureus* (Gram+), *Pseudomonas aeruginosa*, and *Escherichia coli* (Gram-), during a simulated hemoperfusion session. This preliminary in vitro study, simulating in all respects clinical practice, has the objective of clarifying the binding affinity of Gram+ and Gram- bacteria, present contextually as well as in the clinical condition of interest, and, at the same time, evaluating the filter ability to break down the simulated bacterial load to have a reference value of filter “binding ability” carrying out this clinical study on sepsis-affected patients.

For this purpose, a kinetic study was carried out, evaluating, for each time point, the bacterial counting by fluorescence-activated cell sorting (FACS) analysis and the detection of the number of colony-forming units (CFUs) by selective culture media. Finally, to verify the CFUs attached to the filter, the latter was divided into three sections, and a portion of the stationary phase was cultured on selective media for the three investigated bacterial strains and subjected to ultrastructure analysis by scanning electron microscopy (SEM).

## 2. Materials and Methods

### 2.1. Microbial Strains and Preparation of the Culture Suspension

The following bacterial strains from the University of Messina’s in-house culture collection (Messina, Italy) were used for the preparation of the microbial suspension: *Staphylococcus aureus* ATCC 6538, *Escherichia coli* ATCC 10536, and *Pseudomonas aeruginosa* ATCC 9027. The overnight starter cultures were grown in Tryptic Soy Broth (TSB, Oxoid, CM0129) at 37 °C for 24 h and washed three times in filtered phosphate-buffered saline (PBS) by centrifugation at 3500× *g* for 10 min. The bacterial suspensions were then diluted to a density of approximately 10^8^ CFU/mL, as spectrophotometrically recorded at 560 nm (Shimadzu UV-1601, Kyoto, Japan), and inoculated by syringe into a sterile bag containing 1000 mL of a 0.9% NaCl solution.

### 2.2. In Vitro Simulated Adsorption with Seraph^®^-100 and Experimental Design

**Figure 2:**
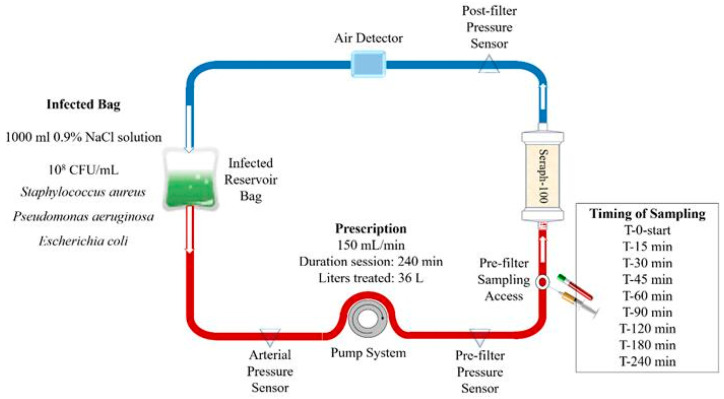
Schematic overview of the experimental set-up.

A schematic overview of the experimental setup is given in Figure 2.

A Seraph^®^-100 disposable broad-spectrum absorbent device for extracorporeal blood purification (ExThera Medical, Martinez, CA, USA), consisting of ultra-high molecular weight polyethylene microspheres coated with heparin/heparan sulfate, was attached to a renal replacement therapy (RRT) device (Multifiltrate, Fresenius Medical Care AG & Co. KGaA, Bad Homburg, Germany), establishing a closed circuit. A flow rate of 150 mL/min was applied, and the Seraph^®^-100 was pre-treated with a 500 mL 0.9% NaCl solution, according to the manufacturer’s instructions. After the filling of the entire circuit, determining a negligible extracorporeal volume of 100 mL, the reservoir bag containing the bacterial suspension (see previous section for specifications) was added to the closed circuit, starting the perfusion, which was then continued for 4 h (150 mL/min). The reservoir bag was periodically shaken during the procedure to ensure that the mixture of bacteria and samples was taken at the pre-filter level before the starting time (0 min) and at defined time points during the session (15, 30, 45, 60, 90, 120, 180, and 240 min). The experiment was repeated three times, and a microbiologist analyzed all samples in a blinded manner. A circuit with the same setup without the Seraph^®^-100 filter was used as a control.

### 2.3. Bacterial Cell Counting

#### 2.3.1. Fluorescence-Activated Cell Sorting (FACS) Analysis

The FACS technique was used to quantify the three bacterial populations simultaneously. In the specific case, since the objective of this analysis was to count the CFUs present regardless of the specific bacterium present, to evaluate the reduction of the total bacterial load, only the light scattering technique, based on the deviation of the light beam based on the physical characteristics of the particles, was chosen. Two informative parameters were collected: forward scatter (FSC), which provides information on the size of the analyzed particles, and side scatter (SSC), to determine the graininess, roughness, or nucleus/cytoplasm ratio. The graph that can be obtained from this simple analysis is a one-dimensional cytogram or a two-dimensional dot-plot, where each dot represents a single cell detected and analyzed. This analysis, conducted against a specific reaction blank, allows for the evaluation of the reduction of the charge during the treatment, setting the bacterial count of the original bacterial solution to 100% to calculate the percent removal. FACS analyses were performed using a clinical flow cytometry system (BD FACS Canto™, Milan, Italy) according to the manufacturer’s instructions.

#### 2.3.2. Detection of Bacterial Strains by Selective Culture Media

For bacterial strain (*S. aureus*, *E. coli,* and *P. aeruginosa*) detection and counting, 50 µL of each time point of the kinetic experiment (0, 15, 30, 45, 60, 90, 120, 180, and 240 min) were sown in selective culture media: Baird Parker agar (BP), Tergitol TTC agar (TTC), and *Pseudomonas* CN agar (CN), respectively. Cultures were incubated for 48 h at 37 °C.

#### 2.3.3. Evaluation of the Bacteria-Binding Capacity

To evaluate the bacteria-binding capacity of the filter and its possible selectivity for a specific bacterial strain, at the end of the experiment (240 min), the reservoir bag, the filter, and the waste bag were analyzed in triplicate by a blinded microbiologist. Specifically, 50 µL of the reservoir and waste bag content was plated directly into the selective agar culture media (BP, TTC, and CN), whereas the filter was cut under sterile conditions into three sections: head (H), body (B), and tail (T). The content of the three filter sections was recovered and used to prepare suitable suspensions (5 mg/mL), which were also seeded (50 µL) in the selective agar culture media (BP, TTC, and CN). Cultures were incubated for 48 h at 37 °C. Finally, to verify that the bacteria had effectively adhered to the surface of the filter stationary phase, samples of the three filter sections were also investigated through SEM analysis with a Zeiss EVO MA10 (Carl Zeiss S.p.A., Milan, Italy) at an acceleration voltage of 20 kV. Filter stationary phase H, B, and T samples (1 mg) were fixed in 70% ethanol for 48 h and dehydrated through an ethanol series (90% and 100%, 1 h each). Samples were mounted on stubs (SEM-PT-F-12), covered by conductive adhesive tables, and left at 28 °C for 12 h, avoiding the critical drying point, before being covered with 20 nm gold palladium.

### 2.4. Statistical Analyses

Three independent experiments in triplicate (*n* = 3) were carried out. The results, expressed as n. CFU as a function of time (min), were analyzed using a non-linear regression approach based on the three-parameter sigmoid equation followed by the Shapiro–Wilk test using SigmaPlot 12.0 software (Systat Software Inc., San Jose, CA, USA). Data were considered statistically significant for *p* < 0.05.

## 3. Results

All experiments were performed under stable conditions without technical problems, and the cartridge was perfused without interruptions.

To evaluate the filter’s ability to break down the bacterial load and the specific binding affinity of the three bacterial strains towards the heparin-coated beads, an in vitro simulated perfusion with three reference strains, one Gram+ (*S. aureus*) and two Gram- bacteria (*P. aeruginosa* and *E. coli*), each 10^8^ CFU/mL, suspended in a 0.9% NaCl solution, was carried out by Seraph^®^-100 according to the manufacturer’s instructions.

**Figure 3:**
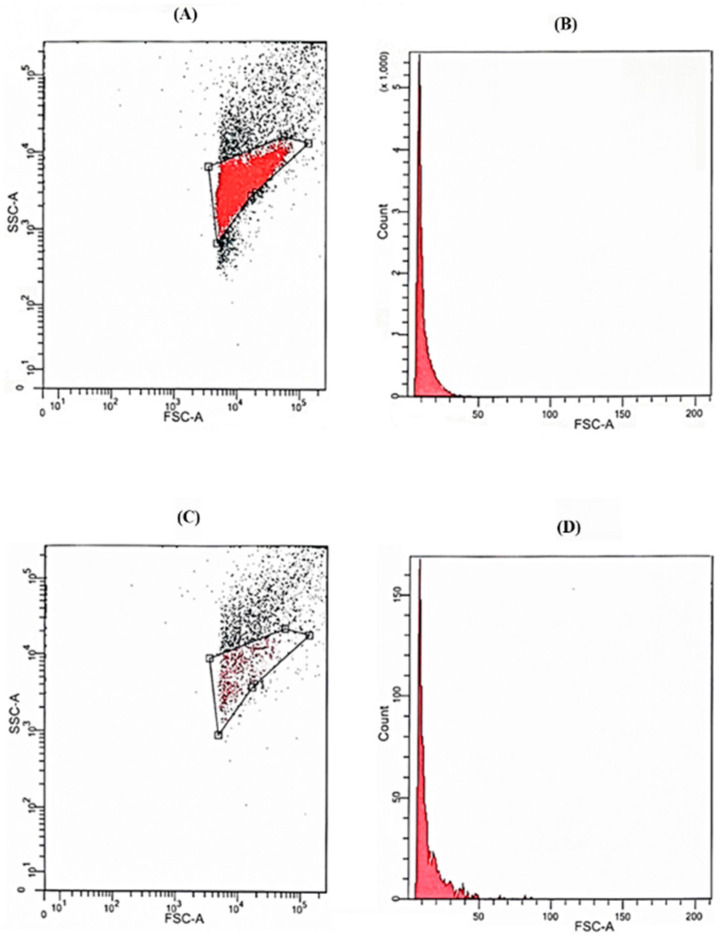
Two-dimensional dot-plot and one-dimensional cytogram of bacterial cell counting obtained by FACS analyses at the starting point (T_0_, (**A**) and (**B**), respectively) and at the end of the simulated in vitro perfusion session (T_240 min_, (**C**) and (**D**), respectively) with Seraph^®^-100.

The analyses of kinetics points (T_0–240 min_) by the FACS technique allowed for calculating the total reduction of the bacterial load at the end of the treatment as being equal to 78.77% (7.88 × 10^7^ CFU/mL). The two-dimensional dot-plot and one-dimensional cytogram of bacterial cell counting obtained by FACS analyses at the starting point (T_0_, Figure 3A and Figure 3B, respectively) and at the end of the simulated in vitro perfusion session (T_240 min_, Figure 3C and Figure 3D, respectively) with Seraph^®^-100 are depicted in Figure 3.

**Figure 4:**
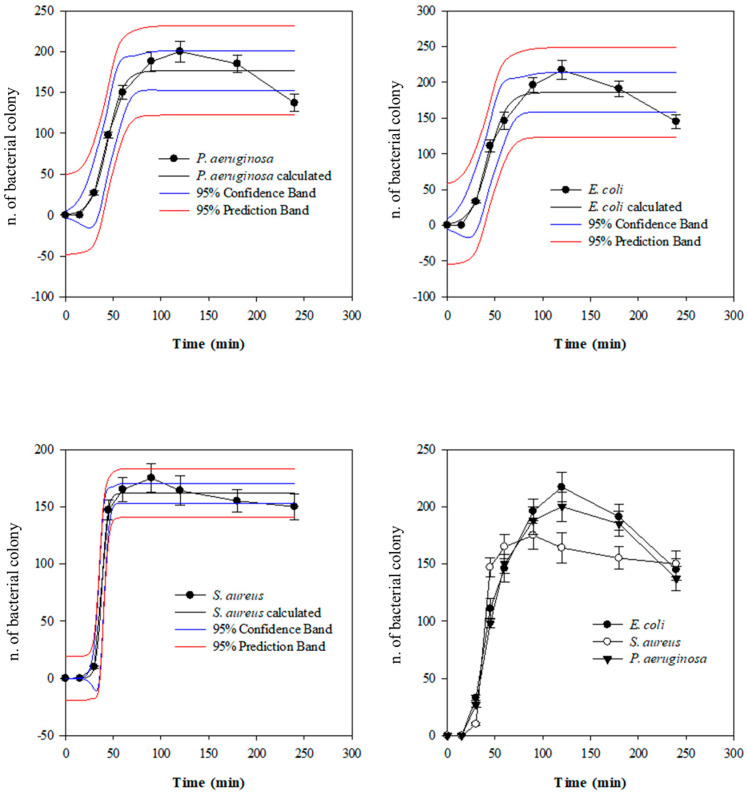
Non-linear regression analysis of kinetic experiments (T_0 – 240 min_). Results, which represent the average of three independent experiments in triplicate (*n* = 3), were expressed as the n. of colony-forming units (CFUs) with respect to time (min), as detected by selective culture media.

The load reduction recorded at the various times also allows for clarifying, with absolute certainty, that the maximum filter sequestration of the bacterial strains already occurs in the first 30 min of treatment, as confirmed by the plate count carried out on selective media for the three investigated bacterial strains (Figure 4).

The results, expressed as n. CFU as a function of time (min), were analyzed using a non-linear regression approach based on the three-parameter sigmoid equation reported below:

$$y=\frac{a}{1+e^{-\left(\frac{x-x0}{b}\right)}}$$

where

*a* = upper asymptote
*b* = slope (growth rate)
*x*0 = crossover point (time of maximum growth)

In Figure 4, beyond the growth curve of each investigated bacterial strain (*P. aeruginosa*; *E. coli*; *S. aureus*), confidence (blue lines) and predicted bands (solid black lines) were depicted. The first ones were used to represent the uncertainty in estimating a curve based on limited or noisy data, while the prediction band was used to represent the uncertainty in the value of a new data point on the curve subject to noise. As can be seen from the three panels of Figure 4, the growth curves fall perfectly within the confidence bands and follow a trend that is almost superimposable to that of the predicted band. This demonstrates the reliability of the recorded data.

Table 1 shows the comparison of the parameters used for the non-linear regression analysis for each investigated bacterial strain and the Shapiro–Wilk normality test, used to evaluate the statistical significance of the results recorded for each kinetic data point depicted in Figure 4 for each investigated bacterial strain.

**Figure 5:**
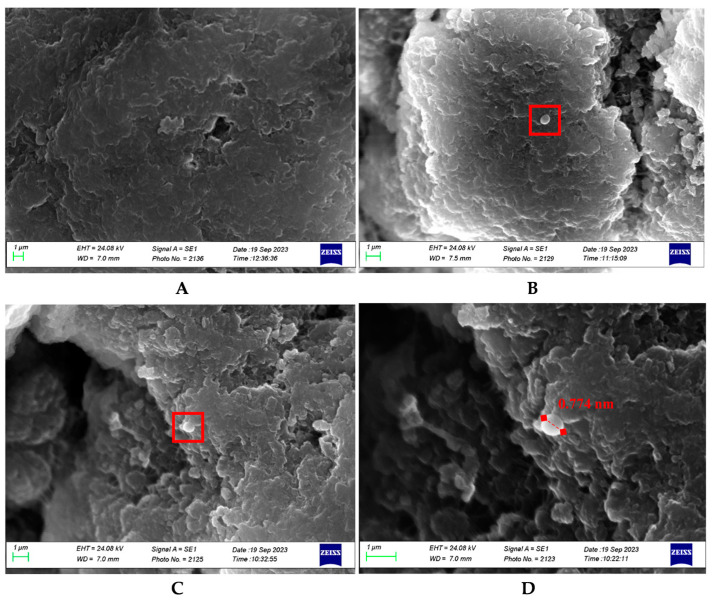
Representative SEM micrograph of the blank stationary phase (heparin/heparan sulfate-coated beads) of Seraph^®^-100 (**A**) in comparison with the Seraph^®^-100 stationary phase at the end of perfusion time (4 h). (**B**,**C**) show some bacteria (red rectangle) attached to the filter stationary phase; (**D**) shows a magnification of (**C**), in which the bacterial size is highlighted.

As can be seen from Figure 4, in which it is possible to observe an overlap of the three growth curves of the three investigated bacterial strains, and from Table 1, the slope or growth rate of *S. aureus* (Gram+) is significantly lower than the other two investigated bacterial strains, both Gram−. This demonstrates a greater affinity of Gram+, and in this case of *S. aureus*, for Seraph^®^-100, which translates into a greater sequestration rate of this bacterial strain during the simulated in vitro perfusion procedure. However, it is worth underlining that the efficiency of reducing the bacterial load of the three investigated strains individually by counting them on selective media reveals a very efficient ability of the filter to sequester the three investigated bacterial strains with reductions equal to 99.96%, 99.85%, and 99.87%, i.e., leading to a final bacterial load of 4 × 10^4^ CFU/mL, 1.5 × 10^5^ CFU/mL, and 1.3 × 10^5^ CFU/mL vs. the starting bacterial load of 1 × 10^8^ CFU/mL for *S. aureus*, *E. coli*, and *P. aeruginosa*, respectively. These data, recorded on the samples (T_0–240 min_) taken during the kinetic experiment, were further validated by the analyses conducted on the filter sections: H, B, and T. Indeed, to experimentally verify whether the bacteria remained bonded to the heparin/heparan sulfate-coated beads, the filter was sectioned into three parts, and suspensions of representative aliquots of the stationary phase of each section were prepared. Knowing the weight of the entire stationary phase, the n. CFU was calculated (H + B + T), with an average value of about 1 × 10^8^ for *S. aureus*, *E. coli*, and *P. aeruginosa*, respectively, according to the above results. Furthermore, this experiment also allowed for verifying the distribution of bacteria within the stationary phase of the filter, which are located approximately 50% in filter H and the remaining 50% equally between filters B and T. The binding affinity of the bacterial strains to heparin/heparan sulfate-coated beads was also corroborated by SEM analysis, as depicted in Figure 5.

## 4. Discussion

This study demonstrated that Seraph^®^-100 adsorbed three bacteria from a super-infected solution during an in vitro simulated hemoperfusion session, suggesting a new era in treating bloodstream infections caused by several pathogens, often inducing sepsis. A bacterial load is related to the severity of sepsis and increased mortality [28,29,30]. Seraph^®^-100 removed 10^8^ CFU/mL of *S. aureus*, *E. coli*, and *P. aeruginosa* contemporary inoculated into a 1000 mL 0.9% NaCl solution, a concentration higher than 100 CFU/mL commonly observed in adult patients with bacteremia [31].

Interestingly, we noted a greater affinity of Seraph^®^-100 towards the Gram+ *S. aureus* when comparing the kinetic data of Gram- pathogens such as *E. coli* and *P. aeruginosa*.

**Figure 6:**
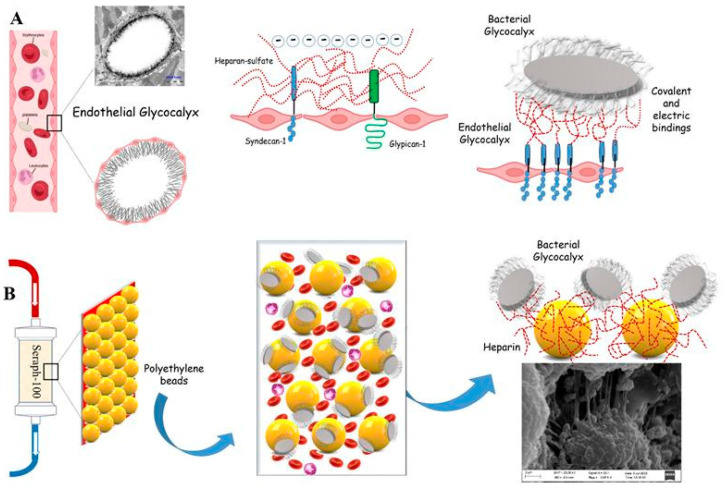
Adsorption processes in Seraph^®^-100 heparin-coated microspheres, which mimic the heparin sulfate of the endothelial glycocalyx. (**A**) The endothelial glycocalyx and bindings of bacteria. (**B**) Seraph structure miming the endothelial glycocalyx.

Electrical and chemical bonds mediate this adsorption through coated microspheres with immobilized heparin, which mimics the heparan sulfate (HS), a type of sulfated glycosaminoglycan of the endothelial glycocalyx, which is targeted by several different pathogens as an initial attachment site during their pathogenesis [32]. This allows for the formation of a negative electrostatic barrier that separates negatively charged blood components, such as red blood cells, and, at the same time, catches elements with opposite electric charges and through specific ligands, often localized in the bacterial glycocalyx (Figure 6).

These challenges between bacteria, endothelium, and immune cells occurred similarly within Seraph^®^-100, and probably the thinner peptidoglycan mesh surrounding Gram- cells, compared to the thicker layers of peptidoglycan and glycocalyx observed in *S. aureus*, could support the different absorption underlined by our results. At the same time, the similar chemical structure of peptidoglycan in Gram+ and Gram− bacteria could also explain the non-selective effects of Seraph^®^-100 on bacteria, viruses, and fungi [33]. However, these bonds are not mediated exclusively by HS, as observed in *P. aeruginosa*, which could interact with the cells through specific poly-cationic ligands [34] or in *E. coli*, whose attachment to heparin-coated polyethylene beads depends on fimbrial adhesin expressions [35,36].

Recently, Seffer revealed the ability of this filter to adsorb *S. aureus* using miniaturized and engineered Seraph^®^-100 adsorbers through pre-treated micro-columns with an infected 0.9% NaCl solution and infected human plasma [25].

Conversely, our data refer to an in vitro model applying to a circuit of hemoperfusion, the commercialized Seraph^®^-100 filter, which was stressed by the crossing of 36 L of a super-infected solution, miming clinical practice, and not a laboratory test. Our analyses revealed specific kinetic adsorption models for each bacterium and assessed the reduction of more than 90% of bacterial CFUs, not only for *S. aureus* but also for *E. coli* and *P. aeruginosa*. These data strengthen the already-published ones, highlighting the ability of this adsorber to remove several pathogens [20].

However, whereas these studies analyzed a single-pass removal of a single bacterium through the filter or its miniaturized columns, we tested the commercialized filter, revealing that the contemporary presence of the three bacteria did not influence their single adsorption, considering Seraph^®^-100 potentially effective also in patients with superinfections as often observed in clinical practice.

Behind these kinetic data, this study evaluated the adsorption properties of Seraph^®^-100, analyzing the distribution of the attached bacteria throughout the filter, underlying that the head was the principal site of adsorption, with less attachment recorded in the body and the filter tail. This distribution, confirmed by SEM analyses, suggests an early saturation of the first area of Seraph^®^-100 and, associated with the peak of bacterial CFU reduction after 30 min, hypothesizes a time-dependent adsorption mechanism and a strong bond between bacteria and heparin-coated beds. This latter concept is essential for the safe use of Seraph^®^-100, explaining the absent back-release of attached pathogens from the filter to the bloodstream after their irreversible adsorption [23].

These data allow for considering Seraph^®^-100 as different from other blood filters often used in septic patients, acting on a precocious etiological level of the sepsis cascade and preventing it through the direct removal of the infective agents from the bloodstream. Conversely, other adsorber devices and hemofilters applied to hemoperfusion or renal replacement therapies reduced cytokines or endotoxins. According to these results, a combined therapy could be a new approach in bacteremic patients, based on a precocious treatment with Seraph^®^-100 associated in series with highly permeable/high cut-off membranes for the prevention of sepsis, eliminating pathogens from the bloodstream, and removing inflammatory mediators, with the possibility of breaking the “cytokine storm”. Moreover, dead bacterial cells or fragments of their cell walls, as observed during the antibiotic treatment, may induce inflammation and harmful dysregulated host responses, whose elimination from the bloodstream could increase host tolerance and bactericidal mechanisms by the innate and acquired immune systems [37].

However, further studies should confirm in vivo the efficacy of Seraph^®^-100 in infected patients, trying to solve significant unmet needs, such as the timing of intervention. Clinical trials are required to assess clinically relevant endpoints such as the incidence of sepsis, hospital length of stay, and mortality. The challenge is also to test this filter in series with other devices, trying to hit more pathways that are over-expressed in a critically infected patient.

At the same time, antimicrobial dose optimization in patients undergoing CRRT is challenging, and despite its growing use in critically ill patients, the paucity of pharmacokinetic data during CRRT limits evidence-based antibiotic dosing recommendations for novel agents. Further data are required to evaluate the efficacy and clinical application of adsorber filters [38,39].

In vitro studies revealed that Seraph^®^-100 did not remove several antibiotics, but further in vivo studies should evaluate and confirm if a drug dose adjustment is required, considering its non-selective adsorption process and avoiding sub-therapeutic antibiotic levels, which negatively influence the success of sepsis treatment and overall survival.

This study has several limitations. The experimental model was based on analyses conducted in a hemoperfusion circuit associated with the commercialized filter but analyzed the adsorption effects of inoculated pathogens in a saline solution, not comparable to the human whole blood. For this reason, potential interactions between bacteria, blood components, such as cells and proteins, and Seraph^®^-100 could not be investigated. Furthermore, the absence of concomitant antibiotic therapy should be underlined.

However, this study simulated clinical practice, demonstrating the effectiveness of this treatment.

## 5. Conclusions

Seraph^®^-100 represents an efficient device to remove, through adsorption, *S. aureus*, *E. coli*, and *P. aeruginosa*, suggesting its precocious use during bacteremia or superinfection. Further studies should confirm in vivo the efficacy of Seraph^®^-100 in infected patients, highlighting that the complexity of sepsis cannot be faced and solved only through a single device but by applying different therapeutic strategies, for example, the combined use of Seraph^®^-100 and hemofilters. A multidisciplinary team should design clinical trials to overcome the limitations of this study.

## Figures and Tables

**Table 1:** Comparison of the sigmoid parameters of the three investigated bacterial strains.

Sigmoid Parameters	*E. coli*	*S. aureus*	*P. aureginosa*
a	185.856	161.812	176.860
b	9.386	2.986	8.227
X0	43.319	38.135	43.799
Normality Test (Shapiro–Wilk) P/F *			
	P	P	P

* P = pass; F = fail. The Shapiro–Wilk normality test was used to evaluate the statistical significance (*p* < 0.05) of each data point of the kinetic curve of each investigated bacterial strain.

## Data Availability

The data underlying this article will be shared on reasonable request to the corresponding author.
